# Emerging T-cell lymphomas after CAR T-cell therapy

**DOI:** 10.1038/s41375-025-02574-x

**Published:** 2025-04-07

**Authors:** Till Braun, Florian Kuschel, Kristin Reiche, Maximilian Merz, Marco Herling

**Affiliations:** 1 Department I of Internal Medicine, Center for Integrated Oncology (CIO) Aachen Bonn Cologne Düsseldorf, Translational Research for Infectious Diseases and Oncology (TRIO), University Hospital Cologne, Cologne, Germany; 2 Mildred Scheel School of Oncology Aachen Bonn Cologne Düsseldorf (MSSO ABCD), Cologne, Faculty of Medicine and University Hospital of Cologne, Cologne, Germany; 3 Center for Scalable Data Analytics and Artificial Intelligence (ScaDS.AI), Dresden, Leipzig, Germany; 4 Institute for Clinical Immunology, University Hospital of Leipzig, Leipzig, Germany; 5 Fraunhofer Institute for Cell Therapy and Immunology IZI, Leipzig, Germany; 6 Cancer Center Central Germany (CCCG) Leipzig-Jena, University Hospital of Leipzig, Leipzig, Germany; 7 Department for Hematology, Cellular Therapy, Hemostaseology, and Infectious Diseases, University Hospital of Leipzig and Comprehensive Cancer Center Central Germany (CCCG), Leipzig/Jena, Germany; 8 Myeloma Service, Memorial Sloan Kettering Cancer Center, New York City, NY USA

**Keywords:** Translational research, Cancer genetics, T-cell lymphoma

## Introduction

Chimeric antigen receptor (CAR) T-cell therapies have transformed the treatment landscape for relapsed/refractory (r/r) B-cell non-Hodgkin lymphoma (B-NHL) and multiple myeloma (MM), delivering unprecedented response rates even in heavily pretreated patients [1, 2]. However, their remarkable efficacy does not come without risks. While acute toxicities such as cytokine release syndrome (CRS) and immune effector cell-associated neurotoxicity syndrome (ICANS) are well-documented, a new and alarming concern has emerged. In November 2023, the U.S. Food and Drug Administration (FDA) reported 22 cases of T-cell malignancies in patients treated with CAR-T cell therapy [3], placing secondary T-cell lymphoma on the growing list of potential complications of CAR-T cell therapy. Of particular concern are cases of CAR-expressing T-cell lymphomas, raising urgent questions about whether vector integration events during CAR-T cell manufacturing disrupt gene expression and contribute to malignant transformation. In this perspective, we integrate the latest evidence on CAR+ T-cell lymphomas, dissect their diagnostic as well as clinical features, and explore the molecular mechanisms that may drive their emergence. We further discuss the potential clinical implications of these findings and strategies to mitigate this emerging risk.

## Clinical spectrum of CAR+ T-cell lymphomas

Before addressing the clinical spectrum of CAR+ T-cell lymphomas, it is essential to first establish a clear definition of these malignancies. To distinguish a CAR+ T-cell lymphoma from a physiological CAR+ T-cell expansion, specific criteria must be met: *(i)* autonomous and uncontrolled T-cell proliferation, leading to a clinical manifestation, *(ii)* proof of clonal T-cell expansion, and *(iii)* an elevated mutational burden characterized by gain-of-function (GOF) mutations in oncogenes or loss-of-function (LOF) alterations in tumor suppressor genes. Additionally, *(iv)* an aberrant immunophenotype of CAR+ lymphoma populations compared to physiologically expanded CAR T-cell can further support the diagnosis of a CAR+ T-cell lymphoma. Establishing these diagnostic criteria is critical for differentiating true malignant transformation from benign, therapy-related T-cell expansions, to ensure appropriate clinical management.

To assess the relative risk of CAR-T-associated lymphomas, several academic centers have conducted long-term follow-ups of their patients. A notable example is the work from Stanford University, where investigators analyzed 724 patients who received cellular therapies, the majority of whom had undergone CAR-T therapy [4]. With a median follow-up of 15 months, only a single case of T-cell lymphoma was identified, which turned out not to carry the CAR transgene. In addition, the French DESCAR-T registry reported just one case of T-cell lymphoma among 3066 CAR-T-treated patients, this time with confirmed CAR integration [5]. Using a different approach, an analysis of the FDA’s Adverse Event Reporting System found that T-cell lymphomas accounted for 3.2% of all secondary malignancies following CAR-T therapies [6]. Collectively, these data suggest that T-cell lymphomas, and in particular CAR+ T-cell lymphomas, constitute a rare but notable complication of CAR-T cell therapy, with an incidence estimated to be well below 1%.

To date, detailed clinical and molecular characterizations at different levels of granularity for ten cases of CAR+ T-cell lymphomas have been published (Table 1*,* see Supplementary Table 1 for a methodological assessment). Two of these cases originated from a first-in-human trial in which CD19-directed CARs were generated from allogeneic T cells using the piggyBac transposon system for the treatment of relapsed lymphoma [7]. Given that the incidence of CAR+ T-cell lymphoma in this trial reached 20%, further clinical development of piggyBac-based CAR-T products was discontinued. While no definitive evidence of insertional mutagenesis was found in these two cases, a potential contribution of the piggyBac-based approach to the development of CAR+ T-cell lymphomas appears likely, possibly due to high levels of DNA breaks induced by the high-voltage electroporation used in the transposon system.

**Table 1:** Clinical spectrum of CAR+ T-cell lymphoma.

Indication	CAR-T product	Time point	Clinical presentation	T-cell phenotype	T-cell clonality	Genomic CAR integration site	Mutational burden	Treatment (best response)	Classification	Reference
r/r DLBCL	CD19-specific CAR T-cells^a^ (piggyBac)	Sixteen months	Nodal	CD4^+^ TEMRA cells	Yes	24 integration sites *(*e.g*. FYN, LOC107985043, BACH2)*	*PIGA*	glucocorticoids, cyclo, and vincristine (PR)	Aggressive T-cell lymphoma, NOS	*Micklethwaite* et al., *Blood, 2021*
r/r B-ALL	CD19-specific CAR T-cells^a^ (piggyBac)	Twelve months	Nodal	CD3^-^ CD8^+^ T cells	Yes	4 integration sites *(e.g. LOC107985043, BACH2)*	n.a.	unknown chemo + allo-SCT (CR)	Aggressive T-cell lymphoma, NOS	*Micklethwaite* et al., *Blood, 2021*
r/r primary central nervous system lymphoma	tisa-cel (lentiviral)	One month	Non-nodal, association with HLH	CD4^-^CD8^-^ T cells	Yes	*DPF2, RAB11FIP3, NPLOC4*	*TET2, DNMT3A*	glucocorticoids, toci, cylo, and eto (PR)	n.a.	*Kobbe* et al., *NEJM, 2024*
r/r DLBCL	tisa-cel (lentiviral)	Three years	Cutaneous	CD4^+^ T cells	Yes	*PLAAT4*	n.a.	PUVA, BV, and gemcitabine (PR)	Primary cutaneous CD30+ T-cell lymphoma	*Dulery* et al., *Nat Med, 2025*
r/r multiple myeloma	cilta-cel (lentiviral)	Nine months	Cutaneous and intestinal	CD8^+^ EM T cells	Yes	*KPNA4, ZPGAT, polycomb-associated ncRNAs*	*TET2, JAK1, PRR5L*	glucocorticoids (PR)	T-LGLL-like	*Braun* et al., *Nat Med, 2025*
r/r multiple myeloma	cilta-cel (lentiviral)	Four months	Intestinal	CD4^+^ T cells	Yes	*SSU72*	*CXCR1, PRKD3*, *MAP2K3*	MMF (PD); cyclo (PR)	Indolent T-cell lymphoma of the GI tract	*Ozdemirli* et al., *NEJM, 2024*
r/r multiple myeloma	cilta-cel (lentiviral)	Five months	Nodal and cutaneous	CD4^-^CD8^-^ T cells	Yes	*PBX2*	*TET2, NFKB2, PTPRB, JAK3*	CHOEP (CR); GDP+alemtuzumab + allo-SCT (PR); tofacitinib (PD); radiatio + peg-IFN + ECP (CR)	Aggressive T-cell lymphoma, NOS	*Harrison* et al., *NEJM, 2025*
r/r multiple myeloma	cilta-cel (lentiviral)	Sixteen months	Nodal and cutaneous	CD4^-^CD8^-^ T cells	Yes	*ARID1A*	*TET2*	CHOEP (CR)	Aggressive T-cell lymphoma, NOS	*Harrison* et al., *NEJM, 2025*
r/r multiple myeloma	cilta-cel (lentiviral)	Four months	Intestinal	CD8^+^ T cells	Yes	*n.a*.	*SH2B3*	budesonide, glucocorticoids, and infliximab (PR); ustekinumab (PD); CSA (PR)	Indolent T-cell lymphoma of the GI tract	*Hosoya* et al., *ASH, 2024*
r/r multiple myeloma	cilta-cel (lentiviral)	Two months	Intestinal	CD4^+^ T cells	Yes	10 integration sites, most prominently in *TP53, TANGO2*	*SOCS1, DNMT3A*	glucocorticoids, tacrolimus, infliximab (PD), ruxolitinib (PR)	Indolent T-cell lymphoma of the GI tract	*Perica* et al., *NEJM, 2025*

Summary of clinical and genomic information on ten cases of CAR+ T-cell lymphomas that have been published to date.

*DLBCL* diffuse large B-cell lymphoma, *HLH* hemophagocytic lymphohistiocytosis, *cilta-cel* ciltacabtagen-autoleucel, *tisa-cel* tisagenlecleucel, *EM* effector memory, *TEMRA* terminal effector memory T cells, *CR* complete remission, *PR* partial remission, *PD* progressive disease, *MMF* mycophenolate mofetil, *cyclo* cyclophosphamide, *CSA* ciclosporin A, *toci* tocilizumab, *BV* brentuximab vedotin, *allo-SCT* allogeneic stem cell transplantation, *GDP* gemcitabine, dexamethasone, cisplatin, *peg-IFN* pegylated interferon alfa-2a, *ECP* extracorporeal photopheresis, *GI* gastrointestinal, *NOS* not otherwise specified.

^a^Derived from HLA-matched sibling donor.

Six additional cases have been reported in patients treated with Ciltacabtagene autoleucel (cilta-cel) for r/r MM [8–12] and two more cases treated with tisagenlecleucel (tisa-cel) for r/r B-NHL [5, 13]. Although the absolute number of CAR-T-treated patients in MM is lower than in r/r B-NHL, cilta-cel currently appears to be associated with the highest reported incidence of CAR+ T-cell lymphomas. This may be related to the increased mutational burden in MM patients, who often have a history of extensive and prolonged prior treatments, including high-dose alkylating agents [14].

The immunophenotype of the predominantly mature, malignant T cells in these secondary lymphomas has varied across reported cases, encompassing CD4^+^ [5, 9, 15] and CD8^+^ variants [8, 11] as well as CD4^-^CD8^-^ manifestations [10, 12, 13]. Clinically, CAR+ T-cell lymphomas have demonstrated not only nodal involvement, but also distinct predilection to extranodal sites, particularly the skin [5, 8, 10] and the gastrointestinal system [8, 11, 15]. Notably, with the exception of the two cases described by Harrison et al. [10, 12], most patients who developed CAR+ T-cell lymphomas following commercial CAR-T cell therapy exhibited a relatively indolent disease course. These malignancies frequently emerged at the intersection of autoimmunity and malignancy, as exemplified by their association with hemophagocytic lymphohistiocytosis (HLH) in the case reported by Kobbe et al. [13]. However, based on their proliferative behavior, clonality, and mutational burden, these lymphomas qualified as overt malignancies rather than benign lymphoproliferations. Importantly, while rare cases of aggressive CAR+ T-cell lymphomas responded to polychemotherapy [12], indolent cases often demonstrated favorable responses to immunosuppression by glucocorticoids [5, 8] or ciclosporin A [11].

Notably, attempting to classify the reported cases within the existing WHO 2022 framework [16] underscores the remarkable heterogeneity of their clinical presentations. These range from T-large granular lymphocyte leukemia (T-LGLL)-like manifestations [8] to indolent T-cell lymphomas of the gastrointestinal tract [9, 11, 15] and, in rare instances, aggressive peripheral T-cell lymphomas [10]. However, as these iatrogenic events occur under non-sporadic circumstances, current definitions are insufficient to comprehensively categorize these entities, necessitating an adjustment in forthcoming WHO classifications, as previously demonstrated with the recognition of breast implant-associated anaplastic large cell lymphoma (BIA-ALCL).

## Pathogenetic concept of CAR+ T-cell lymphoma

Surveying the genomic landscape of reported CAR+ T-cell lymphomas, *TET2* LOF aberrations emerge as a recurrent feature, detected in four of seven examined cases involving a commercial CAR-T product [8, 10, 12, 13]. A particularly illustrative example is our reported case [8], in which we characterized a biclonal process, tracing the clonal evolution of a *TET2*-mutated precursor from a monoallelic LOF mutation to loss of heterozygosity through *TET2* deletion. TET2 has previously been identified as a critical regulator of CAR+ T-cell proliferation, serving as a safeguard against uncontrolled expansion. In a murine model, biallelic *TET2* loss enabled antigen-independent proliferation of CAR-T cells, driven by sustained expression of the AP-1 factor BATF3 and an MYC-dependent proliferative program [17]. In agreement, *TET2* disruption due to CAR transgene integration into one *TET2* allele, combined with an additional hypomorphic *TET2* mutation in the other allele, enhanced the therapeutic efficacy of CD19-directed CAR-T cells in a reported case [18]. Notably, *TET2* is a highly recurrently mutated gene in clonal hematopoiesis of indeterminate potential (CHIP), a condition characterized by the age-related expansion of hematopoietic clones carrying mutations in genes involved in epigenetic regulation [19], such as additionally *DNMT3A*, which was also found to be mutated in two CAR+ T-cell lymphoma cases [13, 15]. While the development of CAR+ T-cell lymphomas appears to be rooted in CHIP-related alterations, additional oncogenic events are likely required to drive full malignant transformation. Among these additional oncogenic events, mutations in *JAK* family members [8, 10] and JAK/STAT regulating genes [15], as well as defects in DNA damage regulators, such as *CHK2* [8], have emerged as potential contributors. These alterations may further enhance proliferative signaling and impair genomic integrity, creating a permissive context for malignant progression in the background of CAR-T cell therapy.

Based on the recurrent observation of pre-existing CHIP mutations, our current assessment of these CAR-T+ lymphomas departs from insertional mutagenesis as the primary driver of these secondary neoplasms. This perspective is supported by the identification of a highly heterogeneous landscape of CAR integration sites (Table 1). However, this does not entirely exclude insertional mutagenesis as a contributing factor to malignant transformation. Notably, a mono-allelic CAR vector integration into *TP53* has been reported in a single case, marking the only published instance of a CAR+ T-cell lymphoma with integration into a well-established cancer-associated gene leading to reduced expression of the respective gene product. Similarly, CAR transgene integration in regulatory regions of *TET2*, leading to its biallelic disruption without malignant progression, highlights how such events can alter T-cell behavior without necessarily driving transformation [18]. Further investigations are needed to clarify the oncogenic potential of specific integration events, particularly the monoallelic disruption of *TP53*, which was also observed in a CAR-T patient cohort without evidence of malignant progression [20].

**Fig. 1:**
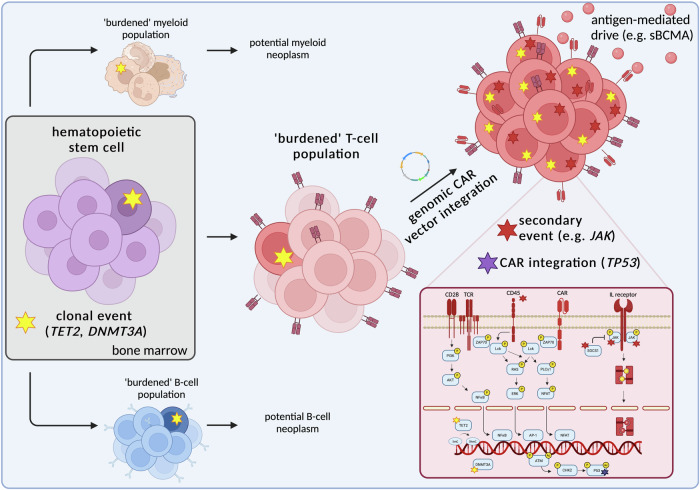
Proposed pathogenetic model of CAR+ T-cell lymphomas. Suggested trajectories towards CAR+ T-cell lymphoma in the context of pre-existing clonal hematopoiesis of indeterminate potential (CHIP) based on published cases. A mutation in an epigenetic regulator, most commonly *TET2* or *DNMT3A* (yellow asterisk), arises at low frequency in the hematopoietic stem cell compartment. This CHIP-associated alteration leads to a ‘burdened’ myeloid and B-cell lineage, the latter of which potentially even giving rise to the initial B-cell malignancy for which the CAR-T cell therapy was intended. Simultaneously, these precursor lesions can create a permissive T-cell compartment. Following genomic integration of the CAR vector, selective expansion of the mutated T-cell clone occurs, driven by persistent antigenic stimulation, potentially through the CAR’s own target, such as soluble BCMA (sBCMA). The CAR itself thus functions as a continuous signaling driver [24], promoting uncontrolled T-cell proliferation. Additionally, secondary genomic events (red asterisk), such as mutations in *JAK* or *MAPK* signaling pathways, further enhance pro-survival and proliferative signaling. Disruptions in DNA damage regulators, exemplified by *CHK2*, may contribute to genomic instability, facilitating full malignant transformation. To date, genomic CAR integration into a well-established cancer-associated gene has been demonstrated in a single case of CAR+ T-cell lymphoma, in which a monoallelic insertion into the *TP53* gene potentially contributed to malignant transformation. Created in BioRender (https://BioRender.com/f50a476).

In addition to genomic aberrations, we propose that from the moment of transduction, the CAR itself functions as a persistent signaling driver, directly fueling the expansion and survival of the pre-malignant T-cell clone. By delivering continuous activation signals, the CAR may override physiological T-cell regulatory circuits, disrupting the inter-clonal balance that typically ensures controlled T-cell expansion and retraction. This sustained TCR-like input likely provides a selective advantage to a genetically primed clone, enabling perturbation of such normal T-cell homeostasis. An overview of our current pathogenetic concept is provided in Fig. 1.

## Current methodological limitations

In addition, multi-modal molecular analyses are essential for determining the origins of secondary CAR+ T-cell lymphomas. However, among the ten reported cases, none provide a comprehensive molecular characterization across all three critical time points, *(i)* prior to or at the time of apheresis, *(ii)* in the final CAR T-cell product, and *(iii)* following CAR T-cell therapy (see Supplementary Table 1 for an overview of molecular characterizations in each case), limiting our current pathogenetic understanding. Exemplarily, molecular analyses of the final CAR T-cell product were reported in only five cases [7, 12, 13], with just one occurring outside a clinical trial [13]. Due to legal constraints, residual CAR T-cell products are generally unavailable for diagnostics at most clinical centers, representing a major limitation that hinders a deeper understanding of the molecular mechanisms driving secondary CAR+ T-cell lymphomas. Given the rarity and severity of these adverse events, securing access to remnants of CAR T-cell products, particularly from cases reported in the post-authorization phase of the CAR T-cell product life cycle, is crucial for advancing molecular insights into their pathogenesis. To address this limitation, we propose that a minimal set of accompanying diagnostics should include the detection of genomic structural variants, TCR clonality analysis, comprehensive immunophenotyping with a harmonized panel of antibodies, and integration site analysis at all three time points.

## Clinical implications and consequences

Although CAR+ T-cell lymphomas are rare, most patients do not present with obvious masses or radiologic abnormalities, which complicates early detection and clinical diagnosis. Consequently, clinicians and pathologists must maintain a high degree of suspicion when encountering unexplained symptoms suggestive of T-cell infiltration, such as cutaneous manifestations and diarrhea. Prompt recognition of these signs is essential for ensuring timely intervention and appropriate management of CAR+ T-cell lymphoma.

The emergence of CAR+ T-cell lymphomas raises critical questions about risk stratification before CAR-T therapy. One evolving concern is a CHIP-screening prior to CAR-T-cell therapy. While this could theoretically identify individuals at higher risk, it would also lead to the exclusion of approximately 10% of patients [21]. Given the effectiveness of CAR-T cell therapies and the risk of CAR+ T-cell lymphoma being well below 1%, such an approach is hardly justifiable. A second consideration is whether integration site analysis should be implemented before CAR-T infusion. However, the aforementioned highly heterogeneous landscape of observed vector integration, alongside the fact that genomic CAR integration into a cancer-associated gene leading to altered expression has been implemented as central in only one of ten published cases [15], argues against this. Requesting this step would prolong manufacturing timelines, delaying treatment access without substantial benefit. Rather than restricting CAR-T eligibility, new strategies should be explored to enhance treatment safety. First, systematic collection of further cases will allow the establishment of molecular risk models, for better hazard prediction with the least consequence of closer monitoring. For patients who developed a CAR+ T-cell lymphoma, personalized strategies such as ex vivo drug screenings should be explored, allowing for therapeutic interventions based on the specific vulnerabilities of the malignant clones.

Furthermore, the implementation of vectors with inherent molecular safety switches could provide a controlled mechanism to eliminate malignant CAR+ T-cell clones if such arise [22]. Importantly, these approaches could also open new avenues for utilizing naturally occurring T-cell mutations to enhance CAR-T cell therapies [23], ensuring both efficacy and safety.

## Supplementary information

Supplementary Table 1
